# Combined Aerobic Exercise and Virtual Reality-Based Upper Extremity Rehabilitation Intervention for Chronic Stroke: Feasibility and Preliminary Effects on Physical Function and Quality of Life

**DOI:** 10.1016/j.arrct.2022.100244

**Published:** 2022-11-11

**Authors:** Ryan E. Ross, Emerson Hart, Ewan R. Williams, Chris M. Gregory, Patrick A. Flume, Christina M. Mingora, Michelle L. Woodbury

**Affiliations:** a Ralph H. Johnson Veterans Affairs Health Care System, Research Service, Charleston, SC; b Department of Health Sciences and Research, Medical University of South Carolina, Charleston, SC; c Department of Medicine, Medical University of South Carolina, Charleston, SC; d Divison of Occupational Therapy, Medical University of South Carolina, Charleston, SC

**Keywords:** Exercise, Rehabilitation, Stroke, Upper extremity, Virtual reality

## Abstract

**Objectives:**

To (1) examine the feasibility of combining lower extremity aerobic exercise (AEx) with a virtual reality (VR) upper extremity (UE) rehabilitation intervention and (2) provide an estimate of effect size for the combined intervention on UE function, aerobic capacity, and health-related quality of life.

**Design:**

Single-group feasibility trial.

**Setting:**

Research laboratory.

**Participants:**

Community-dwelling individuals with mild to moderate impairment of the UE at least 6 months post stroke (N=10; male, n=6; female n=4; mean age, 54 years).

**Intervention:**

All participants received 18 sessions over a nominal 2-3 sessions per week schedule of a combined AEx and VR-UE rehabilitation intervention. During each session, participants completed 15 minutes of lower extremity AEx followed by playing a VR-UE rehabilitation game for approximately 20 minutes.

**Main Outcome Measures:**

Feasibility was evaluated by metrics of adherence, retention, treatment acceptability, data completeness, and adverse events. UE function, aerobic capacity (peak oxygen consumption [Vo_2_peak]), and quality of life were assessed with the Fugl-Meyer Assessment of Upper Extremity (FMA-UE), expired gas exchange analysis, and Stroke Impact Scale (SIS), respectively.

**Results:**

Adherence was 100%, and there were no withdrawals or losses to follow-up to report. Participants completed the intervention in 49±14 days. Cohen's *d_z_* effect size calculations indicated the intervention elicited medium effects on FMA-UE (*d_z_*=0.50) and SIS memory domain (*d_z_*=0.46) and large effects on absolute Vo_2_peak (*d_z_*=1.46), relative Vo_2_peak (*d_z_*=1.21), SIS strength (*d_z_*=1.18), and SIS overall recovery domains (*d_z_*=0.81).

**Conclusions:**

Combining lower extremity AEx and VR-UE rehabilitation appears feasible in the clinical research setting. Fifteen minutes of lower extremity AEx performed at vigorous intensity appears to elicit clinically meaningful benefits in chronic stroke. Further examination of the combination of lower extremity AEx and VR-UE rehabilitation and its effects on physical function and quality of life is warranted.

With a surviving cohort approaching 8 million individuals, stroke is the leading cause of long-term disability in the United States.^1^^,^^2^ Approximately 60% of survivors of chronic stroke report having unmet needs related to impairments of body functions, while 50% report unmet needs related to limitations and restrictions of activities and participation.^3^ The prevalence of poststroke disability coupled with declining stroke mortality rates^1^ reflects an increasing need to develop effective rehabilitation strategies aimed at reducing disability and improving quality of life for the millions of survivors, their families, and care partners.^4^

Many survivors of stroke experience upper extremity (UE) hemiparesis,^5^ which contributes to functional limitations and reduced quality of life.^6^^,^^7^ Reducing UE impairment remains a top priority of survivors of stroke^8^ and rehabilitation clinicians. Virtual reality (VR)-based UE rehabilitation may offer significant, moderate improvements in body functions and activity outcomes compared with conventional therapy.^9^^,^^10^ Additionally, VR-UE rehabilitation is unique in that it can provide real-time feedback, can provide expert action observation, and has the potential to be deployed for telerehabilitation and patient-driven home use.^11^ While VR-UE rehabilitation is a promising and novel intervention,^12^ therapies designed to improve a single aspect of stroke recovery may be limited in their effectiveness because of the presence of other stroke sequelae. Therefore, to effectively address the heterogenous nature of chronic stroke, multimodal interventions must be developed.

Aerobic exercise (AEx) is widely known for its overall health benefits.^13^^,^^14^ There is growing support for including AEx in standard of care stroke rehabilitation because of its ability to improve aerobic capacity^15^ and potential to influence other domains of stroke recovery. Recent evidence indicates that AEx combined with UE repetitive task practice can enhance UE motor recovery,^16^ walking capacity,^17^ and health-related quality of life^18^ in survivors of chronic stroke. Aerobic exercise can enhance neuroplasticity^19^^,^^20^ and physical capacity,^21^ both of which are vital components of stroke recovery. This highlights the potential for AEx to serve as an ideal adjunct for stroke rehabilitation interventions. Furthermore, adapting newer technologies and investigating multimodal interventions have been identified as areas of needed research.^22^ Thus, combining AEx and VR-UE rehabilitation is warranted but, as far as we know, has yet to be explored. Therefore, the overall purposes of this study were to (1) examine the feasibility of combining AEx with a VR-UE rehabilitation intervention and (2) provide an estimate of effect size for the combined intervention on UE function, aerobic capacity, and health-related quality of life.

## Methods

This study was approved by and followed the ethical standards of the Medical University of South Carolina Institutional Review Board (ClinicalTrials.gov Identifier: NCT04259424; Trial registration date: February 6, 2020). Prior to performing any experimental procedures, all participants provided written informed consent.

### Participants

Ten community-dwelling survivors of stroke were enrolled between March 2021 and January 2022. Participants were initially screened from a stroke registry database to identify candidates and limit the potential of screen failures. Inclusion criteria were as follows: (1) age 21-90 years; (2) experienced unilateral stroke at least 6 months but no more than 120 months prior; (3) voluntary shoulder flexion of the affected arm ≥20° with simultaneous elbow extension ≥10°; (4) mild to moderate arm movement impairment (Fugl-Meyer Assessment of Upper Extremity [FMA-UE]> 21 but <54 points); (5) passive range of motion in paretic shoulder, elbow, wrist, thumb, and fingers within 20° of normal; (6) ability to communicate as per the therapists’ judgment at baseline testing; and (7) ability to complete and pass an exercise tolerance test. Exclusion criteria were as follows: (1) lesion in brainstem/cerebellum because these may interfere with visual-perceptual/cognitive skills needed for motor relearning; (2) presence of other neurologic disease that may impair motor learning skills; (3) orthopedic condition or impaired corrected vision that alters reaching ability (eg, prior rotator cuff tear without full recovery); (4) history of, or current, depression; (5) unable to understand or follow 3-step directions; (6) severe cognitive impairment (Montreal Cognitive Assessment [MoCA] score <20); (7) severe aphasia; (8) inability to read English; (9) history of congestive heart failure, unstable cardiac arrhythmias, hypertrophic cardiomyopathy, severe aortic stenosis, angina, or dyspnea at rest or during activities of daily living; and (10) severe hypertension with systolic >200 mmHg and diastolic >110 mmHg at rest. All participants completed a cardiopulmonary exercise test (CPET) and received physician clearance prior to initiating the study intervention.

### Outcome measures

Outcome measures were assessed at the beginning of study procedures and within 1 week of the final intervention session for all study participants.

### UE impairment and function

UE impairment was assessed with the FMA-UE motor recovery.^23^ The FMA-UE is a reliable and valid measure of motor impairment^24^, ^25^, ^26^ and the recommended outcome measure for trials targeting motor function in chronic stroke.^27^ The FMA-UE consists of 33 items; however, the 3 items testing reflex response were not administered because they do not measure a voluntary movement construct.^28^ Therefore, item ratings were summed and reported out of 60 points, with larger numbers indicating greater UE motor ability. The FMA-UE was administered by a licensed occupational therapist, video recorded, and scored by a trained rater who was blinded to the assessment time point.

UE functional ability was assessed with the Wolf Motor Function Test (WMFT).^29^ The WMFT has been demonstrated to be a reliable and valid measure of motor function in chronic stroke.^24^^,^^30^ The WMFT contains 15 movement tasks and a grip strength assessment. The 15 movement tasks are scored in 2 ways. First, the time required to complete each task was recorded in seconds, with lower scores meaning faster speed/more efficiency while completing each task. Second, quality of hemiparetic UE movement was rated on the Functional Ability Scale, a 6-point rating scale (eg, 0: does not attempt tasks with hemiparetic UE; 5: movement of the hemiparetic UE appears to be normal). Grip strength of the hemiparetic UE was assessed with a hand-held dynamometer.

### Health-related quality of life

The Stroke Impact Scale (SIS) version 3.0 is a reliable and valid patient self-report survey that assesses physical function and other dimensions of health-related quality of life: emotion, communication, memory and thinking, social role function, and overall recovery.^31^^,^^32^ Items were summed and converted to a domain score (0-100), with higher scores indicating greater quality of life. The SIS-recovery subtest is a single-item in which the participant rates their perceived poststroke recovery from 0%-100% recovered.

### Cognitive function

Cognitive function was assessed with the MoCA.^33^ The MoCA has demonstrated reliability and validity for assessment of cognitive function in chronic stroke.^34^^,^^35^ The MoCA is a 30-point test that briefly assesses short-term memory recall, attention and concentration, executive function, language, visuoconstructional skills, and orientation.

### Aerobic capacity

Participants completed a CPET on a recumbent cycle with pulmonary gas exchange assessments at pre- and post intervention.^a^ During the CPET, breath-by-breath rate of oxygen consumption (V̇o_2_), respiratory exchange ratio (RER), heart rate, rating of perceived exertion (RPE), and blood pressure were assessed. Participants began by pedaling at ∼15 W at ∼60 revolutions per minute and exercise intensity was increased ∼15 W every 2 minutes until volitional fatigue was reached. Peak oxygen consumption (Vo_2_peak) was determined by the highest 30-sample average of V̇o_2_ during the final stage of the test. Because survivors of stroke do not often meet the physiological criteria needed to establish a maximum V̇o_2_, we used RPE≥17 and/or RER>0.95 as an indicator of maximal effort and Vo_2_peak.^36^

### Feasibility outcomes

To comply with recommendations for feasibility and pilot trials^37^ and stroke rehabilitation research,^38^ data were gathered regarding adherence, retention, treatment acceptability, data completeness, and adverse events. Definitions of each of these are provided in appendix 1.

### Intervention

The intervention was delivered 2-3 times weekly for a total of 18 sessions. At each session, participants received AEx followed by the VR-UE rehabilitation game. Participants were provided a 10-minute rest break between AEx and VR-UE rehabilitation.

### Aerobic exercise

Participants performed 15 minutes of AEx on a recumbent stationary cycle.^b,c^ RPE (Borg 6-20 scale)^39^ and heart rate were assessed prior to, every 5 minutes during, and 5 and 10 minutes post exercise. Exercise intensity was determined using the Karvonen equation.^40^ The target heart rate (THR) of each AEx session was 70% heart rate reserve (HRR) and remained constant throughout the intervention. Participants were instructed to maintain a pedaling cadence ∼70 revolutions per minute, and the resistance was manipulated to achieve the THR.

### VR-based UE rehabilitation

UE rehabilitation was delivered with the Recovr Rehabilitation System.^11^^,d^ The Recovr Rehabilitation System platform houses interactive VR-based games that were deliberately designed to enhance paretic UE movement quality via individualized progressive movement practice along with an array of performance metrics allowing for within-session feedback on movement performance. Participants completed UE rehabilitation sessions by playing the Duck Duck Punch (DDP) game on the Recovr Rehabilitation System platform.^11^ A detailed description of DDP is provided in appendix 2. Pain, heart rate, and RPE were assessed prior to DDP and approximately every 5 minutes or at every 50 DDP targets reached. Pain was assessed using the Numeric Pain Rating Scale where participants rated pain levels of their hemiparetic UE on an 11-point scale (0: no pain; 10: worst pain imaginable).^41^

### Data analysis

Means and SDs of demographic and outcome variables and are presented in Tables 1 and 2. Descriptive statistics for feasibility outcomes are reported. Effect sizes (Cohen's *d_z_*) were calculated for the AEx+DDP intervention on all outcome measures. Pearson correlations between demographic variables (ie, age, body mass index, stroke chronicity), baseline functional assessments (ie, MoCA, FMA-UE, WMFT Functional Ability Scale, grip strength, absolute and relative Vo_2_peak), and change in FMA-UE were performed to explore potential predictors of response to AEx+DDP.

**Table 1:** Sample demographic information

Variable	AEx+DDP (N=10)
	
Sex (female), n	4
Age (y), mean ± SD	53.6±11.5
BMI, mean ± SD	29.6±7.0
Race, n	
Black	6
White	4
Stroke chronicity (mo), mean ± SD	56.0±35.6
Stroke type (ischemic/hemorrhagic), n	8/2
Stroke hemisphere (left/right), n	5/5
Dominant UE affected, n	6

Abbreviation: BMI, body mass index (calculated as weight in kilograms divided by height in meters squared).

**Table 2:** Outcome measures and effect size estimates

Variable	AEx+DDP (N=10) Pre	AEx+DDP (N=10) Post	Post-Pre Mean Change	Effect Size *d_z_*
MoCA	23.8±2.3	24.5±2.8	0.7±1.3	0.55
FMA-UE	32.7±8.5	35.3±9.0	2.6±5.2	0.50
WMFT FAS	2.87±0.6	2.96±0.8	0.09±0.47	0.13
WMFT time (s)	39.1±24.4	39.2±22.9	0.10±17.6	0.02
WMFT grip strength (lb)	15.6±8.1	17.4±8.7	1.8±5.4	0.33
Peak aerobic capacity (mL/kg*min^−1^)	18.1±4.7	20.3±5.4	2.2±1.8	1.21
Peak aerobic capacity (L/min)	1.59±0.4	1.78±0.4	0.19±0.13	1.46
Stroke Impact Scale				
Strength	47.5±21.5	60.0±21.1	12.5±10.6	1.18
Memory	73.9±19.3	80.4±14.8	6.4±14.0	0.46
Emotion	87.8±11.6	86.7±6.3	−1.1±10.1	−0.11
Communication	83.6±15.9	82.5±16.7	−1.1±10.9	−0.10
ADL	78.0±13.6	78.0±14.6	0.0±4.3	0.00
Mobility	77.8±16.1	79.7±16.6	1.9±8.3	0.26
Hand	22.5±21.4	22.0±22.8	−0.5±6.0	−0.08
Participation	70.3±16.9	70.3±17.5	0.0±11.7	0.00
Recovery	51.5±18.6	60.0±18.1	8.5±10.6	0.81

NOTE. Data are presented as mean ± SD.

Abbreviations: ADL, activities of daily living; FAS, Functional Ability Scale.

## Results

### Feasibility

All participants completed pre- and post testing for FMA-UE, WMFT, MoCA, and SIS. Gas exchange data were not obtained for 2 participants; thus, Vo_2_peak data reported are from 8 participants. Adherence was 100% because all participants were able to complete all planned sessions. On average, participants completed 18 intervention sessions in 49 days (range, 39-86 days). Mean pain rating of the hemiparetic UE prior to and after DDP was 1.2±2.0 and 2.1±2.5, respectively, indicating mild pain before and after playing DDP. Mean baseline pain rating of the hemiparetic UE was 1.3±2.1 and 0.9±1.8 on sessions 1 and 18, respectively, indicating that the intervention did not increase resting pain levels. Ratings of perceived exertion were 13.4±1.3 during AEx and 13.9±2.5 during DDP, indicating that participants perceived they were working between “somewhat hard” and “hard” during the intervention. Retention was 100% because there were no withdrawals. There was 1 nonserious adverse event in which a participant sought medical attention while enrolled, although the event was unrelated to trial participation.

### Dose and effects of intervention

#### Aerobic exercise

**Fig 1:**
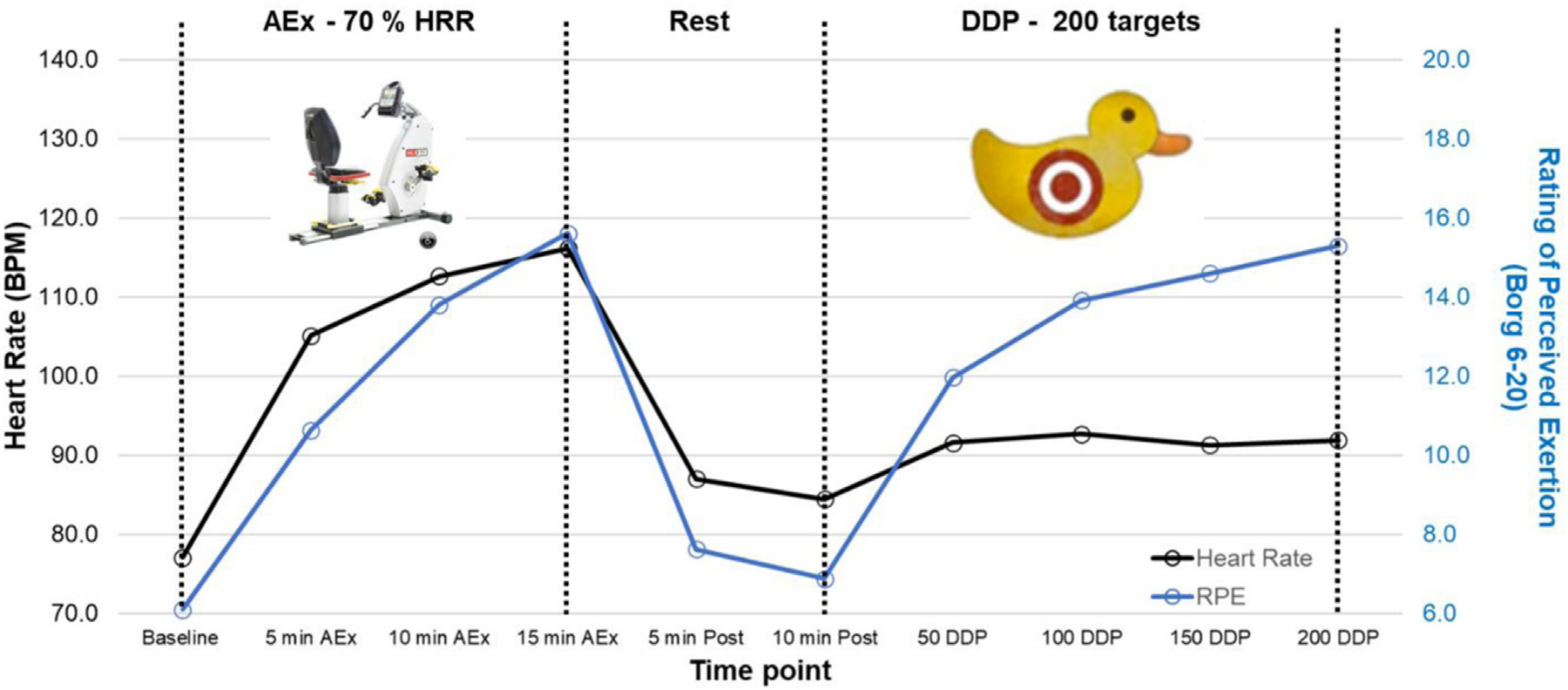
Mean heart rate and rating of perceived exertion response to AEx+DDP. Mean of all 18 sessions for all participants (N=10).

All participants were able to complete all AEx sessions. Mean resting heart rate and peak heart rate achieved during the CPET were 72±12 and 134±12 beats per minute, respectively. During the pre-CPET, participants reached a peak RPE of 18.8±1.4 and peak RER of 1.02±0.06. During the post CPET, participants reached a peak RPE of 19.3±1.4 and peak RER of 1.04±0.11. The mean intensity achieved during AEx sessions was 65±11 %HRR. The mean heart rate and RPE response for all sessions for all participants are depicted in fig 1.

#### VR-based UE rehabilitation

All participants were able to complete all DDP sessions. Mean DDP gameplay time was 22.3±5.8 minutes (not including rest breaks). Participants reached a mean of 185±16 targets per session with a mean success rate of 95%±3%. Although participants were progressed on an individual basis, it took a mean of 4±2 sessions to achieve a 90% success rate on 3 consecutive sessions (indicator to manipulate difficulty level) and 7±4 sessions to successfully reach 200 targets on 3 consecutive sessions (indicator of improving endurance). Participants demonstrated an increase in heart rate while playing DDP, which was equivalent to 33±11 %HRR.

### Preliminary effects of intervention

Absolute changes in outcome measures and effect size estimates are reported in Table 2. Cohen's *d_z_* effect size calculations indicated the AEx+DDP intervention elicited a small effect on WMFT grip strength (mean, 19.2%±34.7%); medium effects on FMA-UE (9.1%±18.4%), MoCA (2.9%±5.2%), SIS memory domain (13.9%±31.1%); and large effects on absolute Vo_2_peak (12.5%±8.1%), relative Vo_2_peak (12.2%±9.6%), SIS strength (44.2%±75.1%), and overall recovery domains (21.6%±26.5%). Stroke chronicity (*r*=−0.47) was moderately associated with change in FMA-UE, and baseline absolute (*r*=0.54) and relative (*r*=0.64) Vo_2_peak were moderately associated with change in FMA-UE. There were no other associations between demographic or baseline functional assessments and change in FMA-UE (all *r*≤0.2).

## Discussion

This study provides preliminary evidence supporting the feasibility of combining AEx and VR-UE rehabilitation, specifically DDP, as a multimodal intervention for chronic stroke. This is supported by strong adherence, participants’ ability to complete all sessions in a reasonable time frame, only 1 adverse event unrelated to study participation, and overall acceptability of the novel intervention. Participants demonstrated a small increase in hemiparetic UE pain after DDP; however, pain ratings remained mild, and participants cited transient increase in local muscular pain as a primary factor for such rating. Baseline pain ratings did not increase throughout the intervention. Participants reported similar levels of perceived exertion during AEx and DDP despite the stark difference in physiological response to both parts of the intervention (∼65%HRR for AEx; ∼33%HRR for DDP); however, the levels of perceived exertion during DDP are comparable with previous work examining exertion during functional task practice in stroke.^42^

Participants successfully reached an average of 185 virtual targets per session. However, the number of reach attempts for each target was greater as participants iteratively tried various UE movement strategies. Such number of movement repetitions per session is comparable with combined AEx and task-practice UE rehabilitation interventions.^16^^,^^43^ We report a medium effect size of the combined intervention on UE impairment as assessed by the FMA-UE. Although the mean change in FMA-UE did not reach the minimal clinically importance difference of ∼ 4-7 points for chronic stroke,^44^ 3 participants exceeded this value, 1 approached this value (4-point change), and the others demonstrated minimal changes. Changes in FMA-UE were moderately associated with baseline relative Vo_2_peak (*r*=0.64) and stroke chronicity (*r*=−0.47), suggesting that survivors of stroke with greater aerobic fitness and/or less time since stroke may have better response to AEx+DDP, although we cannot make definitive conclusions of predictors of response from our limited sample size.

The primary objective of DDP is to promote recovery of forward reaching UE motions, defined as simultaneous shoulder flexion with elbow extension.^11^ DDP does not address wrist/finger recovery. Therefore, we would not expect to see changes in the wrist and hand items of the FMA-UE, 14 of the possible 60 points. Of the mean change of 2.6 points, we found that 2.3 points were gained on nonhand items and 0.3 on hand items. This suggests that DDP is primarily affecting recovery of elbow extension and shoulder flexion. Additionally, as expected, we saw no effect of the combined intervention on WMFT or SIS hand domain given that DDP is targeting recovery at the structure and function level, rather than activity level of the International Classification of Functioning Disability and Health model.^45^ In the future, AEx+DDP could be supplemented with distal motor control training in functional task practice.

The average aerobic exercise intensity achieved, 65 %HRR, is also comparable with previous work in this area by Linder et al.^16^ While the duration of AEx in this trial, 15 minutes, is substantially shorter than other combined AEx and UE rehabilitation trials,^17^^,^^43^ we report large effects of the combined intervention on aerobic capacity (mean increase, 2.2 mL/kg*min^−1^). Keteyian et al reported that with every 1.0 mL/kg*min^−1^ increase in Vo_2_peak, there is an approximate 15% reduction in all-cause mortality in patients with cardiovascular disease; thus, the changes in aerobic capacity demonstrated by participants in this study are clinically meaningful.^46^ Furthermore, prior to AEx+DDP, 37.5% (3/8) of participants were above the threshold (women, 16.5 mL/kg*min^−1^; men, 19.0 mL/kg*min^−1^) for the lowest risk of all-cause mortality, whereas 75% (6/8) exceeded such thresholds after the intervention.^46^ We suspect that such changes are actual changes in aerobic capacity rather than changes in effort during the test, evidenced by comparable RPE and RER values during pre- and postintervention CPETs. A brief but potent bout of 15 minutes of AEx prior to rehabilitation may be ideal for survivors of stroke with limited endurance or elevated levels of fatigue. Additionally, this duration of AEx may be more clinically feasible where time and space may be limited.

AEx+DDP elicited large effects on the strength and overall stroke recovery domains of the SIS, indicating that participants felt that the intervention improved the strength of the upper and lower limbs as well as contributed to their overall recovery from stroke. This is consistent with Rosenfeldt et al who report significant effects of a combined AEx and UE repetitive task practice intervention for chronic stroke on the strength and overall recovery domains of the SIS.^18^ Additionally, AEx+DDP elicited medium effects on cognitive function, as assessed by the MoCA, coupled with medium effects on perceived memory improvement. Both exercise^47^ and VR-UE rehabilitation^48^ have been independently shown to improve cognitive function in stroke; therefore, combining interventions may elicit a synergistic effect on cognition. Furthermore, adding a VR-based cognitive training component to AEx+DDP may enhance the cognitive benefit (real and perceived) of the multimodal intervention.^49^

The mean total time participants spent in active therapy (time completing AEx and playing DDP) was 37.3 minutes per session, although sessions generally lasted ∼1.0 hour including rest breaks and preparatory activities. Given the average actual therapeutic time investment of 11.1 hours over the course of 18 sessions the returns appear to be robust. Per hour of active therapeutic time participants gained, on average, 0.23 FMA-UE points, 0.20 mL/kg*min^−1^ aerobic capacity, 1.13 SIS strength domain points, and 0.77 points SIS overall stroke recovery domain. Such per/therapy-hour gains in these outcome measures are comparable with or exceed other combined AEx and UE rehabilitation paradigms.^16^^,^^18^^,^^43^ AEx combined with VR-UE, and specifically DDP, has the potential to be used in the home, reducing commuting times to and from therapy clinics for patients and improving access to rehabilitation for patients in rural areas. Additionally, the combined intervention can provide increased options for the patient to self-direct movement practice, thereby augmenting and enhancing therapist-directed sessions and potentially reducing costs. Therefore, we suggest that further examination of the combination of AEx and VR-UE rehabilitation and its effects on physical function, quality of life, and cost is warranted.

### Study limitations

Although our results are encouraging, this study does have several limitations. Given our small sample size and the goal of establishing feasibility, the results are not intended to generalize to the larger population with stroke. Furthermore, inclusion for participation in this study was mild to moderate UE impairment; thus, we cannot make conclusions for the effect of the intervention with severe UE impairment. We did not enroll a control group receiving DDP only. Therefore, we are unable to decipher the additive effects of combining these interventions. Additional efficacy work will need to be performed in the laboratory setting before feasibility work aiming to use such intervention in the home of survivors of stroke is commenced.

## Conclusions

Combining AEx and VR-UE rehabilitation appears feasible in the clinical research setting. The combined intervention, AEx+DDP, elicited large effects on Vo_2_peak, perceived strength, and overall stroke recovery and medium effects on UE impairment, cognitive function, and perceived memory in chronic stroke. We report that a relatively short bout of AEx performed at a vigorous intensity can elicit clinically meaningful benefits in chronic stroke. Pairing AEx with stroke rehabilitation interventions may enhance the response to the intervention and simultaneously spark a virtuous cycle of reduced impairment, improved function, and enhanced quality of life. Future work aimed at deconstructing the additive benefits of combining AEx and UE-VR rehabilitation (ie, performing a controlled pilot trial) and the mechanisms by which AEx may enhance response to rehabilitation is needed.

## Suppliers

a. Quark Cardio-pulmonary exercise metabolic analyzer; COSMED. b. Monark 837e; Monark. c. ISO7000R SciFit; SciFit Inclusive Fitness. d. Recovr Rehabilitation System; Recovr Inc.

## Appendix 1 Feasibility outcomes

Adherence was defined as the number of treatment sessions completed divided by the total number of planned treatment sessions. Retention was calculated by the number of participants completing the intervention divided by the number of participants allocated to the group. Treatment acceptability was defined as the willingness and ability of participants to abide by and maintain the treatment schedule. This was assessed by the number of calendar days taken to complete treatments, pain levels before and after treatment sessions, and perceived exertion levels during treatment sessions. Adverse events were defined as unexpected problems that occurred while enrolled in the study. Serious adverse events were defined as events occurring during study participation that led to death, hospital admission, disability, or permanent damage or were life-threatening.

## Appendix 2 Duck Duck Punch description

To play DDP, participants sat in front of a large screen monitor interfaced with a computer, which housed the Recovr Rehabilitation System software, and a Kinect motion sensor. While playing DDP, the participant controls an avatar arm by moving their hemiparetic arm. The Kinect is custom programmed to detect hemiparetic proximal arm motions for forward reach (ie, simultaneously flexing the shoulder and extending the elbow). The participant's physical arm motions are mapped to avatar arm motions so that the participant causes the avatar arm to reach to and “punch” (ie, make contact with) virtual duck targets that appear at various horizontal and vertical locations on the screen. In the game's custom software, trunk motions are constrained by fixing the avatar's shoulder in space. This means that the avatar arm does not respond to participant's attempts to use lateral or forward trunk flexion as a compensatory strategy to overcome challenges with forward reaching. Thus, the participant must use a more typical forward reaching movement strategy as is often the goal of therapist-directed rehabilitation sessions. In other words, a compensatory movement strategy does not result in a successful target hit, thereby eliciting an iterate process where the participant attempts new strategies until a therapeutically beneficial forward-reaching strategy is used to successfully hit the target. Successful game play was motivating and reinforced the movement strategy. The goal of each DDP session was to successfully hit 200 target ducks; however, participants often required multiple attempts to hit a single target, and therefore game play is consistent with evidence-based repetitive task practice interventions. Participants were provided rest breaks after each set of 50 targets, after playing DDP for at least 5 minutes, or if the participant experienced extreme local muscular fatigue. A licensed and experienced occupational therapist adjusted the gameplay parameters to be either more or less difficult according to the participant's ability level (eg, the participant's voluntary shoulder flexion/elbow extension abilities) so that each participant could achieve ∼90% success during the session. Participants who achieved more than a 90% success rate on targets reached on 3 consecutive sessions had their gameplay parameters adjusted to increase the difficulty level.

## Appendix C Supplementary materials

Image, application 1

Image, application 2
