# Supplementary material for: Combined Aerobic Exercise and Virtual Reality-Based Upper Extremity Rehabilitation Intervention for Chronic Stroke: Feasibility and Preliminary Effects on Physical Function and Quality of Life
Source: Arch Rehabil Res Clin Transl. 2022 Nov 11;5(1):100244. doi: 10.1016/j.arrct.2022.100244 (PMC10036233; doi:10.1016/j.arrct.2022.100244)
Supplement: Supplementary file 1 [file mmc1.docx]

Appendix 1. Feasibility outcomes

Adherence was defined as the number of treatment sessions completed divided by the total number of planned treatment sessions. Retention was calculated by the number of subjects completing the intervention divided by the number of subjects allocated to the group. Treatment acceptability was defined as the willingness and ability of participants to abide by and maintain the treatment schedule. This was assessed by the number of calendar days taken to complete treatments, pain levels before and after treatment sessions, and perceived exertion levels during treatment sessions. Adverse events were defined as unexpected problems that occurred while enrolled in the study. Serious adverse events were defined as events occurring during study participation that led to death, hospital admission, disability or permanent damage or were life-threatening.
