# Supplementary material for: Combined Aerobic Exercise and Virtual Reality-Based Upper Extremity Rehabilitation Intervention for Chronic Stroke: Feasibility and Preliminary Effects on Physical Function and Quality of Life
Source: Arch Rehabil Res Clin Transl. 2022 Nov 11;5(1):100244. doi: 10.1016/j.arrct.2022.100244 (PMC10036233; doi:10.1016/j.arrct.2022.100244)
Supplement: Supplementary file 2 [file mmc2.docx]

Appendix 2. Duck Duck Punch description

To play DDP participants sat in front of large screen monitor interfaced with a computer, which housed the RRS software, and a Kinect motion sensor. While playing DDP, the participant controls an avatar arm by moving their hemiparetic arm. The Kinect is custom programmed to detect hemiparetic proximal arm motions for forward reach (i.e., simultaneously flexing the shoulder and extending the elbow). The participant’s physical arm motions are mapped to avatar arm motions so that the participant causes the avatar arm to reach to and “punch” (i.e., make contact with) virtual duck-targets that appear at various horizontal and vertical locations on the screen. In the game’s custom software, trunk motions are constrained by fixing the avatar’s shoulder in space. This means that the avatar arm does not respond to participant’s attempts to utilize lateral or forward trunk flexion as a compensatory strategy to overcome challenges with forward reaching. Thus, the participant must utilize a more typical forward reaching movement strategy as is often the goal of therapist-directed rehabilitation sessions. In other words, a compensatory movement strategy does not result in a successful target hit thereby eliciting an iterate process where the participant attempts new strategies until a therapeutically beneficial forward reaching strategy is used to successfully hit the target. Successful game play was motivating and reinforces the movement strategy. The goal of each DDP session was to successfully hit 200 target ducks, however participants often required multiple attempts to hit a single target and therefore game play is consistent with evidence-based repetitive task practice interventions. Participants were provided rest breaks after each set of 50 targets, playing DDP for at least 5 minutes, or if the participant experienced extreme local muscular fatigue. A licensed and experienced occupational therapist adjusted the gameplay parameters to be either more or less difficult –according to the participant’s ability-level (e.g., the participant’s voluntary shoulder flexion/elbow extension abilities) so that each participant could achieve ~90% success during the session. Participants that achieved more than a 90% success rate on targets reached on three consecutive sessions had their gameplay parameters adjusted to increase the difficulty level.
